# Editorial: Perspectives and opinions in health services, volume II

**DOI:** 10.3389/frhs.2026.1762202

**Published:** 2026-01-20

**Authors:** Andrea Cioffi, Daniel Ślęzak, Farshid Alaeddini, Fernanda Cioffi

**Affiliations:** 1 Department of Clinical and Experimental Medicine, Section of Legal Medicine, University of Foggia, Foggia, Italy; 2 Division of Medical Rescue, Medical University of Gdansk, Gdansk, Poland; 3 Tehran Heart Center, Tehran University of Medical Sciences, Tehran, Iran; 4 Fertilitas Day Surgery Reproductive Medicine, Salerno, Italy

**Keywords:** access to care, equity, health policy, health services, health system

## Introduction

Understanding what shapes the accessibility, effectiveness, and efficiency of health services is a prerequisite for their informed governance. The quality of care depends not only on clinical excellence or the availability of resources, but on how interventions are designed and implemented within specific institutional, economic, and social contexts. At the same time, a system's ability to deliver services effectively requires an equally clear-sighted appraisal of its internal constraints: organizational cultures, governance arrangements, financing schemes, the organization of professional work, and monitoring and evaluation tools. It is at the intersection of these two levels – contextual conditions and the revision of internal arrangements – that the possibility of protecting health in an equitable and sustainable way is ultimately decided.

Against this background, this Research Topic was conceived to gather perspectives and opinions on how to improve the effectiveness, delivery, and efficiency of health services, with the aim of achieving more equitable and sustainable care outcomes. The choice to focus also on argumentative formats such as Perspective and Opinion articles reflects the conviction that, in a phase of accelerated transformation of health systems, producing new evidence is not enough: it is necessary to connect data, theoretical frameworks, and policy choices, making explicit the organizational and ethical implications of different strategies. The 18 contributions collected in this Research Topic are situated within this framework and, drawing on heterogeneous experiences, explore how services can be delivered and optimized to strengthen care quality and distributive justice in health systems.

## Access to essential health services: resource management, prevention, and responses to crises

A first cluster of articles addresses access to health services starting from its most elementary precondition: the availability and continuity of essential functions (1, 2). Zakir et al. highlight that, in public hospitals in Harar, eastern Ethiopia, weak laboratory services translate into repeated interruptions of a substantial share of available tests, including critical investigations for clinical management. These discontinuities are linked not only to shortages of reagents, but to unreliable supply chains and governance arrangements unable to ensure operational continuity, with direct implications for equity and patient safety. Complementing this, Woldeyohanins et al. document, in the comprehensive specialized hospitals of the Amhara region, a substantial proportion of missing or non-functioning equipment, often due to lack of spare parts, delays in installation, and absence of scheduled maintenance – technological investments that risk becoming locked-in capital that does not generate service value.

Donations of medical devices from the so-called Global North do not, in themselves, offer a sufficient solution for low- and middle-income countries. Paustian et al. show that a considerable proportion of equipment donated to two Ugandan hospitals remains unused or fails prematurely because of mismatches with local infrastructure, lack of maintenance capacity, and absence of life-cycle planning. On this basis, the authors formulate eight theses for a sustainability-driven approach to donation that assigns donors responsibility for the entire life cycle of the device and reframes donation not as an episodic charitable gesture, but as a lever to structurally and equitably strengthen recipient health systems.

Beyond technologies and donation policies, access to essential services also depends on how systems invest in prevention, design financing strategies, and organize themselves to withstand sustained pressures, including health crises. Yang et al. document how the progressive implementation of integrated programs for the prevention and control of congenital disorders is associated with a marked reduction in late fetal and early neonatal mortality among infants with malformations, alongside an increase in early fetal mortality and elective terminations of pregnancy, plausibly linked to more timely prenatal diagnosis.

Cao et al. address the sustainability of Pharmacy Intravenous Admixture Services (PIVAS), showing how fragmented tariffs, often set below actual costs, generate a structural tension between the recognized role of these services in ensuring quality and safety and the fragility of their financing model.

Bosonkie et al. analyze the performance of child nutrition services in the Democratic Republic of the Congo during the COVID-19 pandemic, showing how their marginalization within response plans undermined the continuity of a function essential to child health. Acute health crises thus operate as stress tests of systems’ ability to guarantee essential services, revealing structural vulnerabilities that both predate and outlast the emergency (3).

Finally, Alasousi et al. shift the focus to service use patterns in Kuwait, linking high reliance on emergency departments to users’ weak perception of the role of primary care, despite substantial investments in the sector. Patients who identify primary care centers as their first point of contact display significantly greater awareness and trust, while more than half of respondents continue to view the emergency department as more important than the family physician. This clearly indicates that strengthening primary care is not only a matter of infrastructure but also requires information strategies and public legitimation of its coordinating role within the health system.

Taken together, these contributions find an operational synthesis in the 12-step framework proposed by Chen, which organizes a broad and fragmented body of evidence on delays, waiting times, organizational barriers, and social determinants of access into a coherent model that healthcare organizations can use to guide interventions on processes and resources. Access is thus reframed as an object of intentional design, rather than a mere by-product of logistical constraints, in which infrastructure, governance, and social factors must be addressed in an integrated way.

## Resilience, responsibility, and power in health systems

A second thematic axis weaves together theoretical reflection and context-specific analyses around resilience, social responsibility, and the politicization of health policy. Jatobá et al. develop a genuine epistemology of resilience in public health, explicitly distancing themselves from the reductionist use of the term as mere emergency responsiveness (4–6). Building on four axioms, they define resilience as the capacity to guarantee essential functions on a continuous, universal, and equitable basis under external and internal pressures, acute shocks, and chronic stressors. Resilience is not equated with the “coping” ability of individual professionals but conceptualized as a dynamic process of learning and system transformation aimed at preserving universality and equity over time.

Haddiya et al. apply this lens to public nephrology services in ten African countries, asking patients and nephrologists to what extent hospitals can be considered socially responsible – that is, able to ensure quality and appropriateness of care, financial and geographic accessibility, and compliance with ethical and confidentiality standards (7). Their findings depict a stark picture and shift the focus from simple resource scarcity to the quality of governance, financing arrangements, and rights protection, making working conditions, respect for privacy, and effective access to treatment concrete operational indicators of social responsibility.

Perry et al. extend the theme of responsibility to research partnerships in global health, showing how persistent power asymmetries between institutions in the Global North and the Global South continue to shape research agendas, authorship, and the distribution of benefits. The authors call for a structural revision of funding mechanisms, research governance, and authorship criteria so that such programs become sites of epistemic justice rather than reproducing, in updated forms, colonial logics.

Choi and Fitzek bring the focus back inside a single health system by mapping health services research (HSR) in Austria. Their bibliometric analysis documents steady growth in output, concentrated in areas such as mental health, patient-centred care, disease management, and digital solutions, but also reveals gaps concerning vulnerable populations, economic evaluations of primary care, and transparency regarding funding and conflicts of interest. HSR is thus proposed as a form of cognitive infrastructure that is indispensable for informing allocation decisions and organizational reforms.

If the previous contributions address the responsibility of institutions and partnerships in translating principles of equity into practice, similar issues arise when these demands enter national policy arenas. In this regard, Cecannecchia examines the U.S. initiative “Make America Healthy Again” from a combined public health and medico-legal perspective, acknowledging the legitimacy of concerns around chronic disease, environmental determinants, and industrial conflicts of interest, while warning against the risks of a highly politicized agenda grounded in oversimplified causal narratives and selective uses of evidence. The result may be litigation based on weak standards of proof, erosion of the legitimacy of regulatory authorities, and distorted priority-setting, with potentially regressive effects on equity and access: a reminder of the need to keep ambitious policy reforms firmly anchored to robust scientific standards of evidence and rigorous assessments of their broader health and social implications (8, 9).

## Health professions, education, and organizational models for service equity

A third set of contributions centres on health professions, the contexts in which they work, and the organizational models that can either enhance or undermine their role. Senbetu et al. investigate work motivation among nursing staff in nine public hospitals in southern Ethiopia, finding only partially satisfactory levels and marked differences across facilities. Demotivation is attributed primarily to system-level factors – weak leadership, unbalanced workloads, shortages of resources and protective equipment, non-competitive salaries, and limited professional recognition – rather than to individual deficits, underscoring that nurse retention is a structural determinant of the quality and equity of hospital care.

Briguglio et al. shift the focus to chronic care management in Italy, arguing that dietitians and physiotherapists should be regarded as key actors in community-based prevention along the entire continuum of care. Drawing on major orthopaedic care pathways, they illustrate how structured interventions on diet and physical activity, including tele- and home-rehabilitation models, can improve functional outcomes, prevent or delay complications, and reduce reliance on high-intensity treatments.

In the U.S. context, Cheney et al. align with a growing body of work that frames student-run free clinics as strategic infrastructures both for improving access to care among underserved populations and for training future physicians (10, 11). The experience of the Coachella Valley Free Clinic, developed with Latino and Indigenous agricultural worker communities, shows how these settings can function as privileged sites for building structural competency and leadership skills among trainees.

Du Preez et al. reconstruct a values-driven affiliation between a public medical school and a large private provider in Dubai, showing – through the Public Private Affiliation Journey framework – how joint governance, a shared vision, and a non-dominant role for financial incentives can turn a public–private partnership into a stable infrastructure for clinical education, research, and service improvement.

Finally, Bowman et al. analyse, in a Texas safety-net hospital, how patients with systemic lupus erythematosus from underrepresented groups and their clinicians perceive telemedicine. They show that remote visits can reduce important structural barriers, but introduce new inequities related to digital literacy, the quality of the clinical relationship, and language. Telemedicine emerges as a potential lever for equity only if it is intentionally designed around the dimensions of accessibility, acceptability, and appropriateness, rather than adopted as a mere technological substitute for in-person encounters.

## Conclusion

Taken together, the contributions in this Research Topic show that the organization of services is not a self-contained technical layer, but a key locus in which questions of justice, responsibility, and the use of knowledge are worked out in practice. Whether the issue is ensuring continuity of essential services, rethinking resilience as a system property, redefining professional roles and training environments, or interrogating power relations in research and policy, the articles collected here converge on a common point: improving effectiveness, delivery, and efficiency requires simultaneous action on infrastructure, governance, organizational models, and knowledge production. Rather than offering definitive solutions, these papers provide conceptual frameworks, operational tools, and research agendas that can help diverse health systems make their choices more explicit and the consequences of those choices for equity, quality of care, and sustainability more transparent.
